# Evaluation of Therapeutic Strategies to Reduce the Number of Thrombotic Events in Patients With Polycythemia Vera and Essential Thrombocythemia

**DOI:** 10.3389/fonc.2020.636675

**Published:** 2021-02-16

**Authors:** Douglas Tremblay, Heidi E. Kosiorek, Amylou C. Dueck, Ronald Hoffman

**Affiliations:** ^1^ Hematology/Oncology Section, Tisch Cancer Institute, Icahn School of Medicine at Mount Sinai, New York, NY, United States; ^2^ Department of Health Sciences Research, Mayo Clinic, Scottsdale, AZ, United States

**Keywords:** thrombosis, essential thrombocythemia, polycythemia vera, myeloproliferative, surrogate endpoint, study design

## Abstract

Thrombosis is the largest contributor to morbidity and mortality in patients with polycythemia vera (PV) and essential thrombocythemia (ET). Our understanding of the risk factors and pathophysiology of thrombosis in PV and ET patients is developing, including recent insights into the role of aberrant platelet-neutrophil interactions, *JAK2* mutated endothelial cells and the pro-thrombotic inflammatory milieu. To date, few available therapies have demonstrated the ability to reduce the thrombotic burden in patients with these diseases. Although numerous therapeutic agents have been investigated in both PV and ET patients, few studies are designed to assess their impact on thrombotic events. In this review, we first describe the burden of thrombosis in patients with these myeloproliferative neoplasms (MPNs) and briefly explore their pathophysiologic mechanisms. We then critically assess and summarize the evidence behind currently available therapies with attention toward thrombotic endpoints. Finally, we describe a path forward for clinical research in MPNs that involves surrogate endpoint validation, biomarker development, and clinical trial design strategies in order to accurately assess reduction of thrombotic events when evaluating novel therapies.

## Introduction

Polycythemia vera (PV) and essential thrombocythemia (ET) are *BCR-ABL1* negative myeloproliferative neoplasms (MPNs). PV and ET are morphologically distinct, with the former involving an increased red cell mass while the latter is characterized by clonal platelet overproduction (1). However, both PV and ET involve prolonged, relatively indolent courses with the majority of morbidity and mortality attributed to thrombosis. In particular, arterial thrombosis is a hallmark complication of these MPNs. In a large registry of PV patients, an arterial thrombosis was present at or before diagnosis in 16% and venous thrombosis in 7.4% (2). The baseline prevalence of thrombosis is slightly lower in ET, with one series noting 8% and 1.6% of patients with arterial or venous thrombosis, respectively, in the year prior to diagnosis (3). Thrombosis within the splanchnic vasculature, in particular Budd Chiari syndrome, is a characteristic complication in patients with MPNs (4).

The annual incidence of thrombosis after MPN diagnosis is highly dependent on age. In a large study by the Gruppo Italiano Studio Policitemia, patients younger than 40 had a thrombotic rate of 1.8% per year, while patients older than 70 had a rate of 5.1% per year (5). In ET, the rate of arterial and venous thrombosis after diagnosis is approximately 1.2% and 0.6% per year, respectively (6). This incidence of thrombosis is higher than the general population, as demonstrated by a population-based Swedish study where 1-year cumulative incidence was three times and the 5-year cumulative incidence was two times higher in patients with MPNs as compared with the general population. This incidence of thrombosis in both the MPN and general population was highly dependent on age. For instance, in this study, the 5-year cumulative incidence of thrombosis in females age 18–49 was 1.4% and 0.4% in the MPN and general population, respectively, while in females age 70–79, these rates are 9.2% and 2.9% (7).

Our understanding of risk factors associated with PV and ET related thrombosis continues to evolve. Conventionally, high-risk status in PV patients is determined by age 60 or older and/or a prior thrombosis (8). In a study of 1638 PV patients from the European Collaboration on Low-Dose Aspirin in Polycythemia Vera (ECLAP), age greater than 65 was identified as a significant predictor for thrombosis (9). However these and other studies ignore physiologic age and treat chronologic age regardless of a patient’s underlying functional status and comorbidity burden. Prior thrombosis appears to be a potent predictor of subsequent thrombosis both in patients with PV (9) and ET (10).

In ET, the *JAK2V617F* mutation portends a higher risk of thrombosis based on a study of 891 patients. This study developed a prognostic model which is frequently used entitled the International Prognostic Score for Thrombosis in Essential Thrombocythemia (IPSET-thrombosis), which considers age > 60, cardiovascular factors, previous thrombosis, and *JAK2V617F* mutation (10). Patients with a history of thrombosis or those older than 60 with a *JAK2V617F* mutation are considered high risk (11). Interestingly, the degree of elevation of the platelet count has not been shown to be predictive of developing a thrombotic event, although a higher platelet count is associated with bleeding events secondary to developing acquired von Willebrand syndrome (12).

Treatment in both PV and ET involves cytoreduction for high-risk patients, with a number of therapies which will be described in detail. European LeukemiaNet (ELN) response criteria for PV and ET are shown in **Table 1**. These response criteria are used in the majority of clinical trials in PV and ET, however their impact on reducing the incidence of thrombosis is not well established. Some studies have even suggested that response does not have a meaningful impact on reducing thrombotic risk (14).

**Table 1:** European LeukemiaNet (ELN) Response Criteria for Polycythemia Vera and Essential Thrombocythemia (13).

	Polycythemia Vera		Essential Thrombocythemia	
**Complete response**	A	Durable*^a^* resolution of disease-related signs including palpable hepatosplenomegaly, large symptoms improvement*^b^* AND	A	Durable*^a^* resolution of disease-related signs including palpable hepatosplenomegaly, large symptoms improvement, *^b^* AND
	B	Durable*^a^* peripheral blood count remission, defined as Ht lower than 45% without phlebotomies; platelet count ≤400 × 109/L, WBC count <10 × 109/L, AND	B	Durable*^a^* peripheral blood count remission, defined as: platelet count ≤400 ×109/L, WBC count <10 × 109/L, absence of leukoerythroblastosis, AND
	C	Without progressive disease, and absence of any hemorrhagic or thrombotic event, AND	C	Without signs of progressive disease, and absence of any hemorrhagic or thrombotic events, AND
	D	Bone marrow histological remission defined as the presence of age-adjusted normocellularity and disappearance of trilinear hyperplasia, and absence of >grade 1 reticulin fibrosis	D	Bone marrow histological remission defined as disappearance of megakaryocyte hyperplasia and absence of >grade 1 reticulin fibrosis.
**Partial response**	A	Durable*^a^* resolution of disease-related signs including palpable hepatosplenomegaly, large symptoms improvement *^b^* AND	A	Durable*^a^* resolution of disease-related signs including palpable hepatosplenomegaly, and large symptoms improvement, AND
	B	Durable*^a^* peripheral blood count remission, defined as Ht lower than 45% without phlebotomies; platelet count ≤400 × 109/L, WBC count <10 × 109/L, AND	B	Durable*^a^* peripheral blood count remission, defined as: platelet count ≤400 × 109/L, WBC count <10 × 109/L, absence of leukoerythroblastosis, AND
	C	Without progressive disease, and absence of any hemorrhagic or thrombotic event, AND	C	Without signs of progressive disease, and absence of any hemorrhagic or thrombotic events, AND
	D	Without bone marrow histological remission defined as persistence of trilinear hyperplasia.	D	Without bone marrow histological remission, defined as the persistence of megakaryocyte hyperplasia.
**No response**	Any response that does not satisfy partial remission		Any response that does not satisfy partial remission	
**Progressive disease**	Transformation into post-PV myelofibrosis, myelodysplastic syndrome or acute leukemia*^c^*		Transformation into PV, post-ET myelofibrosis, myelodysplastic syndrome or acute leukemia*^c^*	

a Lasting at least 12 week.

b Large symptom improvement (≥10-point decrease) in MPN-SAF TSS.

c For the diagnosis of PV see World Health Organization criteria (WHO); for the diagnosis of post-ET myelofibrosis, see the IWG-MRT criteria12; for the diagnosis of myelodysplastic syndrome and acute leukemia, see WHO criteria.

This review will focus on primarily prevention of thrombosis in PV and ET. However, it is important to note that in patients who have already had a thrombotic event, initiation of anticoagulation is recommended by consensus guidelines (15). The choice of anticoagulation is not specified, but recent evidence from a large, retrospective international study suggests that direct oral anticoagulants (DOACs) have a similar safety and efficacy profile as warfarin (16). Cytoreduction is also recommended after a thrombotic event for prevention of a recurrence. Evidence supporting this practice includes a retrospective study of 1500 MPN patients with a thrombotic event. Cytoreduction with hydroxyurea was associated with a significantly decreased risk of arterial thrombosis recurrence and late, but not early venous thrombosis recurrence. Notably, cytoreduction had no impact on the recurrence of splanchnic vein thrombosis (17).

In this review, we will briefly explore the pathobiology of thrombosis in MPNs and describe currently available agents for treating PV and ET, critically evaluating their efficacy as it related to reduction in thrombotic risk (**Table 2**). Through this examination, we aim to describe the limitations to our present understanding of optimal thrombotic prevention strategies in PV and ET and propose a path forward to answer these questions which have longed plagued this field.

**Table 2:** Summary of available evidence for available therapies for reduction of thrombotic events.

	Thrombosis reduction	Available data	Comments
*Polycythemia Vera*			
Aspirin	Yes	RCT (18, 19) and non-randomized prospective study (9)	Hematocrit was not controlled to be 45%
Phlebotomy <45%	Yes	RCT (20)	
Hydroxyurea	Yes	Prospective study with historical control (21) and non-randomized prospective study (22)	
Interferon	No	N/A	
Busulfan	No	N/A	
Ruxolitinib	No	N/A	Meta-analysis with trend toward reduction (23)
*Essential Thrombocythemia*			
Aspirin	Yes	Retrospective (24)	
Hydroxyurea	Yes	RCT (25)	High-risk patients only, not intermediate risk (26)
Interferon	No	N/A	
Anagrelide	No	N/A	

N/A, not applicable; RCT, randomized control trial.

## Pathobiology

MPN-related thrombosis has a complex and incompletely understood pathogenesis. Hematologic parameters play an important role, with elevations in hemoglobin leading to increased blood viscosity (27). Observations by Pearson and Wetherley-Mein suggested that the degree of hematocrit elevation and incidence of thrombotic events were directly related (28). On a cellular level, interactions between platelet FAS ligand and FAS receptor on red cells result in externalization of red cell phosphatidylserine, leading to assembly of coagulation factor complexes that forms an occlusive thrombus (29). As previously mentioned, the connection between the degree of thrombocytosis and thrombotic events has not been well established. However, platelet aggregation and activation appear to be increased in ET patients as compared with healthy controls (30).

Several lines of evidence suggest other factors contribute to the propensity to develop thrombosis in PV and ET aside from elevations in hematologic parameters. For one, patients who harbor a *JAK2V617F* are at an increased risk of thrombosis even though they lack diagnostic criteria for an overt MPN, so called clonal hematopoiesis of indeterminant potential (31). Multiple groups have also reported that mutated *JAK2* is present in the endothelial cells of some MPN patients, which suggests a common lineage between endothelial cells with hematopoietic stem cells (32–34). In particular, endothelial cells lining the splanchnic vascular bed frequently harbor the *JAK2V617F* mutation in MPN patients (32, 34). Guy et al. developed a murine model where endothelial cells expressed mutated *JAK2*, but not hematopoietic cells. They documented a thrombotic propensity in these mice despite normal hematologic parameters. Utilizing multiple experimental methods, they elegantly demonstrated that *JAK2* mutated endothelial cells promoted leukocyte adhesion and rolling *via* increased surface expression of P-selectin and von Willebrand Factor (35). Neutrophils harboring the *JAK2* mutation have also been shown to be primed to form neutrophil extracellular traps (NETs), which are extracellular strands of decondensed DNA that complex with histones and others neutrophil granular proteins. NETs can ensnare microorganisms and serve as part of the innate immunity (36), but they are also key to the pathogenesis of thrombosis (37). The propensity for *JAK2* mutated neutrophils to form NETs further implicates the central role of leukocytes in MPN-related thrombosis (38).

The proinflammatory milieu in patients with MPN also contributes to their thrombotic propensity. In patients with MPNs, there are increasing levels of circulating cytokines such as interleukin (IL)-2, and IL-6, and tumor necrosis factor alpha (TNF-α) (39, 40). In the previously mentioned *JAK2* mutated endothelial murine model, thrombosis was increased following the administration of tumor necrosis factor alpha (35). Chronic inflammation present in patients with MPNs contributes to accelerated atherosclerosis (41). Elevations in C-reactive protein, a highly sensitive acute phase reactant, in patients with PV and ET is significantly correlated with the occurrence of a major thrombosis (42). Therefore, the etiology of an increased thrombotic potential in patients with MPNs is multifaceted with contributing factors that include increased blood counts, chronic inflammation, *JAK2* mutated endothelium, and aberrant platelet-neutrophil interactions.

## Available Therapies in Polycythemia Vera

### Aspirin

Patients with PV have increased thromboxane synthesis, leading to constitutive activation of platelets that is thought to be a major contributor to their thrombotic propensity (43). Aspirin, a non-selective and irreversible inhibitor of COX-1 and COX-2, decreases thromboxane production and is considered a pivotal aspect of PV anti-thrombosis therapy. However, it’s early adoption was met with some hesitation, based on an early study from the Polycythemia Vera Study Group (PSVG) that demonstrated high dose aspirin (900 mg daily) with dipyridamole led to an increase in gastrointestinal bleeding (44). As a result, aspirin was infrequently used by American clinicians for a period after publication of this study. In a 1999 survey of 1006 providers, only 16.5% of responders indicated using aspiring for the treatment of PV (45). However, evidence from cardiovascular literature suggested that low-dose aspirin was as effective as higher doses with improved tolerance (46). As a result, a low-dose aspirin strategy was explored in a randomized control trial (RCT) embedded in the ECLAP study, which randomized 518 patients to aspirin 100mg or placebo. All included patients had no other indication for or contraindication to aspirin use. Of note, the study was stopped early because of poor accrual; only 28% of the planned accrual (940 patients per group) was achieved. There was a significant reduction in risk of the combined endpoint of nonfatal myocardial infarction, nonfatal stroke, pulmonary embolism, major venous thrombosis, or death from cardiovascular causes (relative risk [RR] 0.40; 95% confidence interval [CI] 0.18–0.91), a secondary endpoint. However, there was no difference in the primary combined endpoint of nonfatal myocardial infarction, nonfatal stroke or cardiovascular death (RR=0.41; 95% CI 0.15–1.15). There was no significant difference in bleeding or mortality. Importantly, generalization of this study is limited as strict hematocrit control was not maintained. During follow up period, the median hematocrit was 46%, with 25% of patients having a hematocrit over 48%. In a subgroup analysis considering only patients with a hematocrit <48%, there was a nonsignificant improvement in the composite endpoint in the aspirin group as compared to placebo (18).

Also embedded in the ECLAP study was a non-interventional, observational component which included 1,638 patients with PV screened for inclusion in the above mentioned ECLAP RCT (n=518 were ultimately enrolled in the RCT portion of aspirin versus placebo) in order to provide a profile of the disease found in routine clinical practice. Of the entire cohort, 955 patients (58.3%) received antiplatelet therapy. Similar to the randomized portion, antiplatelet therapy was significantly associated with a lower risk of cardiovascular events (hazard ratio [HR], 0.72; 95% CI, 0.53 to 0.97). Variability in clinical characteristics, treatment practices and hematocrit control across countries was seen in this observational study. Antiplatelet therapy was not associated with an increased risk of total bleeding (9). A Cochrane meta-analysis of available prospective studies identified two studies [ECLAP and the Gruppo Italiano Studio Policitemia (19)] that demonstrated use of low-dose aspirin, compared with placebo, was associated with a lower risk of fatal thrombotic events, although this benefit was not statistically significant (odds ratio [OR] 0.20, 95% CI 0.03 to 1.14; p = 0.07). Of note, there was insufficient data to analyze venous and arterial thrombosis separately (47). In accordance with the presented data, ELN criteria from 2018 continues to advocate for aspirin therapy for all patients with PV without a history of major bleeding or gastrointestinal intolerance (8).

### Phlebotomy

Therapeutic phlebotomy is the primary method of hematocrit control in PV. Early efforts to determine the optimal hematocrit goal include an analysis of 1638 patients in the ECLAP study who were followed for a median of 2.8 years. There was no difference in the incidence of thrombotic events in the 46-50% and the over 50% groups (48). In the PSVG-01 study, 431 patients were randomized to phlebotomy alone or phlebotomy with chlorambucil or radioactive phosphate (32P). There were more thrombotic events in the phlebotomy alone group versus cytoreductive therapy plus phlebotomy, however there was no association between hematocrit control and thrombosis in a *post-hoc* analysis (49).

The uncertainty in the optimal hematocrit goal approach led to the development of the prospective Cytoreductive Therapy in PV study, which randomly assigned 365 PV patients to a hematocrit goal of less than 45% (low-hematocrit group) or 45%–50% (high-hematocrit group). The study was designed to include 1,000 PV patients to detect a risk reduction of 30% in the low-hematocrit group based on an event rate of 5% per year in the high-hematocrit group. The protocol dictated the hematocrit target which the patient was assigned to had to be maintained during the course of the study (approximately 75% of patients in each group was correctly maintained) and the choice of therapy to achieve that target was left to investigator decision. After 4 years, the study was stopped early because of poor accrual. The primary end point of death from cardiovascular cause or major thrombosis was significantly lower in the more intensively treated group as compared with the less intensively treated (2.7% vs 9.8%, p=0.007). In addition, the primary endpoint plus superficial vein thrombosis occurred in 4.4% of the low-hematocrit group compared with 10.9% of the high-hematocrit group (p = 0.02). There were four and 11 arterial thromboses in the low and high-hematocrit group, respectively. In terms of venous events, there were one and five in the low and high-hematocrit groups (comparative statistics not available) (20). Based on this study, ELN criteria recommend a hematocrit goal of less than 45% in patients with PV (8). Some clinicians advocate for a lower hematocrit goal in female patients (50), taking into account physiologic differences between sexes. However, this practice has not prospectively or retrospectively evaluated.

### Hydroxyurea

Hydroxyurea, a ribonucleotide reductase inhibitor, is the most commonly used front-line agent for cytoreduction in PV. Early studies of hydroxyurea were run through the PSVG and compared 51 patients treated with hydroxyurea to 194 patients who were treated with phlebotomy alone. In patients treated with hydroxyurea, thrombotic events occurred in 9.8% (five patients) compared to 32.8% in the phlebotomy alone in a historical control cohort, a difference which was statistically significant (p = 0.009) (21). Another prospective study run through the French Polycythemia Study Group (FPSG) randomized 292 patients to either hydroxyurea or pipobroman. In the 16 year follow up, there was no difference in thrombosis or hemorrhage between the two groups. However, there was an increased rate of transformation to acute leukemia in patients treated with pipbroman (51).

Additional evidence, albeit derived from non-randomized trials, on the impact of hydroxyurea is from a *post-hoc* analysis of the ECLAP study. Barbui and colleagues selected 1,042 of 1,638 patients who were treated with only phlebotomy or hydroxyurea with phlebotomy to maintain a hematocrit goal of 45%. Utilizing propensity-score (PS) matching to adjust for difference in baseline characteristics between the two groups, there was a significant difference in the number of cardiovascular events (3.0 vs. 5.8 per 100 person‐years) in patients received hydroxyurea versus phlebotomy alone after a median follow up of 2.8 years. However, after stratification by risk category, there was no difference in patients with low-risk disease (age less than 60 and no prior thrombosis), although the event rate in this population (n = 5) limited this analysis. The authors do not also detail the types of thrombotic events in this population (22). While the results of this study suggest that hydroxyurea is beneficial in high-risk PV, there are methodologic concerns with these analyses. The authors of this study used matched sets of one phlebotomy patient and up to two hydroxyurea treated patients with similar PS with replacement in order to maximize sample size (n=1,023 after PS matching), but it is unknown how many subjects were matched more than once. Using replacement for matches (i.e. some subjects may have been matched more than once) results in better “quality” matches and thus decreased bias but increased the variance (standard error) of the estimates. It is recommended when this approach is used to report the distribution of the number of times subjects are used for matching, however this information is not available for this analysis.

It is important to note that there has not been a randomized, controlled trial of phlebotomy plus hydroxyurea versus phlebotomy alone for primary prevention of thrombotic events. Current ELN guidelines strongly recommends cytoreductive therapy, with either hydroxyurea or recombinant interferon-alpha, in high-risk patients or those who are unable to tolerate phlebotomy (8). Hydroxyurea is associated with adverse events, including myelosuppression, oral and leg ulcers, gastrointestinal symptoms, and propensity to develop non-melanomatous skin cancer (52). Thus alternative agents are employed for high risk patients who require cytoreduction.

### Interferon

Recombinant interferon alfa-2 has been a therapeutic option for patients with PV for decades, but its use was hampered by the need for frequent administration and long-term adverse events (53). A pegylated formulation (PegIFNα-2a), allowing for less frequent dosing and improved tolerability, has been explored for treatment of PV in both the first and second line setting. The first trial to examine its efficacy was the PVN1 study, which evaluated 40 patients with PV who were either treatment naïve or had received cytoreductive therapy for less than 2 years. During a median follow-up of 31.4 months, 35 patients (87.5%) achieved a hematologic complete response. There were no thrombotic events during the study period (54). PegIFNα-2a was then evaluated in the second line setting in the Myeloproliferative Disorders Research Consortium (MPN-RC)-111 trial, which enrolled 50 PV patients (in addition to 65 patients with ET) who were refractory or intolerant to hydroxyurea. A complete hematologic response was observed in 22% of patients. Importantly, the cumulative incidence of major vascular events at 1 year was 2% (95% CI, 1%–8%) and at 2 years was 5% (95% CI, 2%–15%) (55).

PegIFNα-2a has also been compared directly to hydroxyurea in the MPN-RC-112 trial, where treatment-naive PV (and ET) patients were randomized to either hydroxyurea or pegIFNα-2a. At 12 months, overall response rate (ORR), which includes both complete response (CR) and partial response (PR), was similar between hydroxyurea and pegIFNα-2a (69.8% and 78.0%, respectively). The rate of thrombosis was not different between the two arms (56). In another randomized trial (DALIAH) comparing pegIFNα-2a versus hydroxyurea, treatment naïve PV patients 60 years or younger were randomized to either pegIFNα-2a or pegylated interferon alfa2b (pegIFNα-2b) and patients older than 60 years old were randomized to 1:1:1 to either pegIFNα-2a, pegIFNα-2b, or hydroxyurea. Of note, this trial also included patients with ET, PMF, and pre-fibrotic myelofibrosis (pre-PMF). After a median follow up of 36 months, the ORR was higher in patients older than 60 with hydroxyurea as compared with pegIFNα-2a or pegIFNα-2b, however there was no difference in complete hematologic response (CHR) (57). This trial has only been presented in abstract form which does not report the effect of treatment on thrombotic complications.

More recently, a monopegylated form of interferon alfa-2b has been developed which allows for dosing every two weeks in contrast to weekly dosing of pegIFNα-2a. Ropeginteferon alfa-2b (ropegIFNα-2b) has been evaluated for the front-line treatment of PV in the phase III PROUD-PV study, which was recently published (58). This trial randomized 257 PV patients who were treatment naïve or received hydroxyurea for less than 3 years to ropegIFNα-2b or hydroxyurea. At 12 months, there was no significant difference in CHR rates between hydroxyurea and ropegIFNα-2b (75% versus 62.1%, p=0.12). In the CONTINUATION-PV study, 95 patients who were enrolled in the ropegIFNα-2b arm continued therapy while 76 patients originally enrolled in the hydroxyurea arm were treated with BAT, which included predominantly hydroxyurea but also conventional pegIFNα-2a. At 36 months, CHR were higher in CONTINUATION-PV patients treated with ropegIFNα-2b (70.5% versus 51.4% p=0.01). In the combined PROUD-PV/CONTINUATION-PV cohort, there was no difference between the ropegIFNα-2b and control group in terms of major cardiovascular events (10% vs. 6%) and major thromboembolic events (3% vs 3%). Details of the thromboembolic events (i.e. location) are not provided in the published manuscript (58). RopegIFNα-2b is approved for the first-line treatment of PV by the European Medicines Agency (EMA) but not yet by the United Stated Food and Drug Administration (FDA).

PegIFNα-2a and ropegIFNα-2b represent promising therapies for PV that are able to induce sustained hematologic and molecular responses, sometimes even after cessation of therapy. Their efficacy in reducing thrombotic burden has not been fully elucidated, however. In addition, there are significant tolerability concerns. In the MPN-RC-111, grade 3/4 adverse events occurred in 39% of subjects and led to discontinuation 13.9% of patients (55). Common toxicities associated with interferon use include fatigue, flu-like symptoms, and injection site reactions. Grade 3 neuropsychiatric and cardiac adverse events occurred in 7% and 4% of patients in one study (59). These toxicities should be carefully considered before initiating therapy and have limited interferons widespread use in place of hydroxyurea.

### Busulfan

Busulfan is an alkylating agent which has been explored as second-line therapy for PV. Evidence to date is more limited for busulfan as compared with hydroxyurea. In a retrospective study of 36 patients with PV or ET, busulfan treatment resulted in a complete hematologic response (CHR) in 83% of treated patients. Of note, this was an elderly population with a median age of 77 years and 33% had a prior thrombotic event. In this study, 6 patients (16.7%) experienced a subsequent thrombotic event, two while on busulfan and the other four after discontinuing this agent (60). Unfortunately, busulfan has been associated with an increased risk of development of leukemia (61). This agent is therefore reserved for elderly patients who require cytoreductive therapy but are intolerant to hydroxyurea and/or recombinant interferon alpha.

### Ruxolitinib

Ruxolitinib, a JAK1/JAK2 inhibitor, is FDA approved for PV patients who are resistant or intolerant to hydroxyurea. In the phase III RESPONSE trial, 222 PV patients with palpable splenomegaly who were intolerant or resistant to hydroxyurea were randomized to ruxolitinib or best available therapy (BAT). Of note, 58.9% of patients randomized to the BAT group were treated with hydroxyurea, despite documented resistance/intolerance. The study met its primary endpoint of hematocrit control and spleen volume reduction. At 32 weeks of follow up, a thrombotic event (portal vein thrombosis) occurred in one patient in the ruxolitnib group (0.9%) versus six (one myocardial infarction, two deep vein thrombosis, one pulmonary embolism, one splenic infarction, and one thrombophlebitis) in the BAT group (5.4%), although this was not a pre-specified endpoint (62). At 80-weeks of follow-up, there were 1.8 and 8.2 thrombotic events per 100 patient-years in the ruxolitinib versus BAT group, respectively (63).

In the RESPONSE-2 trial, 149 PV patients intolerant/resistant to hydroxyurea but without palpable splenomegaly were randomized to ruxolitinib or BAT. There was one (1.4%) thrombotic event in the ruxolitinib group as compared to three (4%) in the BAT group (64). After 80 weeks of follow-up, the rates of thrombotic events per 100 patient-years were 1.5 in the ruxolitinib group, 0 in the ruxolitinib crossover, and 1.9 in the BAT group (65). Given that both RESPONSE and RESPONSE-2 were open-label studies, the RELIEF trial was performed which was double-blind, double-dummy study primarily evaluating symptom improvement. This trial randomized 110 PV patients who were well controlled on hydroxyurea to either ruxolitinib or hydroxyurea. Thrombotic events occurred in two patients (3.7%) in the ruxolitinib group and in two patients (3.6%) in the hydroxyurea group. Of note, more patients in the ruxolitinib group had a history of thrombosis as compared with hydroxyurea (33.3% versus 21.4%) (66).

Given the short follow up time and inadequate sample size of these individual studies, a systematic review and meta-analysis was performed by Masciulli *et al*. (23). Data from four randomized clinical trials were included: RESPONSE, RESPONSE-2, RELIEF, and the MAJIC-PV trial, a randomized phase 2 trial which also included patients with ET but with subanalyses of PV patients available. In the combined dataset (663 patients, 1,057 person-years), 331 PV patients treated with ruxolitinib were compared to 332 patients treated with BAT with a median follow up of 1 year. The overall thrombosis annual incidence rate was 3.09% (95% CI 1.22–4.96) in ruxolitinib treated patients as compared with 5.51% (95% CI 3.72–7.30) in the BAT group, corresponding to a incidence rate ratio of 0.56 (95% confidence interval 0.28–1.11), which did not reach statistical significance (p = 0.098) (23). Therefore, while there is a suggestion that ruxolitinib may reduce thrombotic events in patients with PV, evidence to date is not conclusive and further investigation is needed.

## Available Therapies for Essential Thrombocythemia

### Aspirin

In contrast to PV, aspirin therapy in ET is based solely on observational evidence without evidence from randomized, prospective trials. In a retrospective study of 300 patients with low-risk ET, 198 were treated with antiplatelet monotherapy (95% received low dose aspirin) and 102 were followed with observation alone. A difference in the thrombotic events between the two groups was not observed (21.2 and 17.7 per 1,000 person-years for antiplatelet therapy and observation, respectively, p = 0.60). However, when analysis was restricted to *JAK2V617F* mutated ET patients, there was a significant increase in venous thrombosis (incidence rate ratio [IRR] 4.0; 95% CI: 1.2–12.9; p = 0.02) in the observation group. Similarly, in patients with baseline cardiovascular risk factors a significant increase in rates of arterial thrombosis (IRR: 2.5; 95% CI: 1.02–6.1; p = 0.047) was seen in the observation group (24). Aspirin has also been purported to prevent thrombosis recurrence. In a pooled analysis of three independently reported cohorts 1,500 MPN patients (761 of which were ET) who had a prior arterial or venous thrombosis event within 2 years of MPN diagnosis, antiplatelet therapy was associated with a decrease in recurrent arterial thrombosis (HR 0.49, 95% CI 0.31–0.78, p = 0.003), but not recurrent venous thrombosis (HR 0.71, 95% CI 0.41–1.24, p = 0.24). This study did not report on the subgroup of ET patients (17).

In patients with ET, the accelerated production of platelets with unacetylated COX-1 and COX-2 function could impair the inhibition of thromboxane A_2_ (TXA_2_) biosynthesis with daily aspirin dosing. As such, it has been reported that about 80% of low-dose aspirin-treated ET patients have inadequate suppression of platelet TXA_2_ production based on a small study with comparisons to healthy controls (67). In order to investigate the effects of increased aspirin dosing, a study randomized (1:1:1) 245 patients with ET to either aspirin 100 mg once, twice, or thrice daily in a double-blind fashion. The primary outcome of this study was change in serum thromboxane B2 (sTXB2), which is a biomarker of COX-1 activity and a surrogate end point for efficacy, after two weeks. Urinary prostacyclin metabolite (PGIM) excretion was also measured as a key safety biomarker. Patients who were assigned to the twice-daily and thrice-daily aspirin regimens had significantly lower sTXB2 (reduced by 80%–90% from baseline) as compared with patients randomized to the once-daily regimen along with decreased interindividual variability. Urine PGIM was similar across all three groups. Notably, patients on thrice-daily aspirin had significant increased gastrointestinal disturbances compared with the other groups. Of note, there was no endpoint to evaluate the effect of these various aspirin doses on thrombosis (68). Long-term efficacy, compliance and tolerability of the selected regimen (100 mg twice daily) as compared to 100 mg once daily is ongoing.

Meta-analyses have attempted to conglomerate data on antithrombotic agents in ET, however are limited by the poor-quality data of the assessed studies (47, 69). A recent meta-analysis failed to show any randomized trials in ET patients (69). They identified 24 observational studies, of which 15 had a comparator arm which assessed thrombosis as an endpoint (total of 799 ET patients). Ten of these studies had serious risk of bias and 5 had moderate. Bias in these studies was largely attributed to confounding, patient selection, or deviations from intervention such as crossover. With antiplatelet therapy, of which 80% were low dose aspirin, the relative risk of thrombosis is 0.74 (95% CI 0.29–1.84). Overall, the evidence was rated by the authors as very uncertain (69). In summary, aspirin therapy in ET has an unclear impact on thrombosis. While it is generally recommended that patients with intermediate or higher risk ET should receive aspirin (8), the strength of evidence supporting this suggestion is generally poor.

### Hydroxyurea

Hydroxyurea has been employed in high risk patients, as discussed previously. This therapy was evaluated in a study of 114 high-risk ET patients who had a prior thrombosis or were over 60 years of age. Patients were randomized to hydroxyurea or no myelosuppressive therapy to reduce the platelet count below 600,000/mm^3^. Of note, only 70% of the hydroxyurea group and 69% of the control group were on antiplatelet prophylaxis. After a median follow up of 27 months, two patients (3.6%) in the hydroxyurea group had a thrombotic event (both arterial) as compared with 14 patients (24%) in the control group (11 arterial, three venous), a difference which was statistically significant (25).

Hydroxyurea has also been evaluated in intermediate-risk ET patients. In a study of 382 patients 40–59 years of age without high-risk features, subjects were randomly assigned to receive hydroxyurea plus aspirin or aspirin alone. After a median follow-up of 73 months, there was no significant difference in the composite primary endpoint of time to arterial or venous thrombosis, serious hemorrhage, or death from vascular cause (p=1.0). Of note, the number of thrombotic events were low, with only five and four arterial and venous events, respectively in the hydroxyurea plus aspirin group and seven and three arterial and venous events in the aspirin alone group, respectively (26). Thus, cytoreduction does not have a role for intermediate-risk patients.

Some clinicians target a lower platelet goal for cytoreductive therapy. In fact, ELN response criteria includes a CR as a platelet count of <400 x 10^9^/L, in addition to a leukocyte count lower than 10 x 10^9^/L (13). A retrospective analysis challenges the utility of achieving these strict response criteria. In this study, of the 166 patients with ET treated with hydroxyurea, 134 patients (80.7%) achieved a CR. However, there was no difference in cumulative incidence of thrombosis in patients who achieved a CR versus those who did not (14). In addition, there is no association between platelet count and thrombosis risk, challenging the importance of targeting a specific platelet count with cytoreductive therapy (70). Thus, the clinical importance of achieving an ELN response in ET is uncertain at this time.

### Interferon

Similar to PV, recombinant interferon alfa-2 has been clinically employed to treat patients with ET. In a study of 23 patients with extended follow up (median 14.5 years), recombinant interferon alfa-2 led to a hematologic response in 75% of patients with no thrombotic events developing (71). Contemporary evaluation of pegIFNα-2a includes a single institution phase 2 study of 43 ET patients in the first and second line setting. The reported ORR was 73%. During the 83-month median follow up, 3 ET patients had a major unprovoked thromboembolic event (59). The previously described MPN-RC-111 study included an arm with 65 ET patients. The ORR was 69% and 43% of patients had a CR with pegIFNα-2a treatment. For the entire cohort (including PV patients), the incidence of major vascular events was 2% at 1 year and 5% at 2 years (55). In another retrospective study of 73 ET patients who were treated with interferon alfa-2 (both recombinant and pegylated) with total follow-up of 211 patient-years, there was only 1 thrombotic event (myocardial infarction) consistent with a thrombosis rate of 0.13 per patient and year (72). The MPN-RC-112 study also included a cohort of 81 ET patients randomized to either pegIFNα-2a or hydroxyurea with no difference in thrombosis rates between the two cohorts (including PV patients) (56). Similar safety concerns regarding the use of pegIFNα-2a for ET patients exist as when employed for PV patients. Thus, the use of pegIFNα-2a in the front line is generally restricted to situations where there are concerns about long-terms hydroxyurea use, such as younger patients (age <40) and those who are pregnant.

### Anagrelide

Anagrelide is an oral imidazoquinazoline derivative that decreases peripheral platelet counts by reduction in both megakaryocytic hyperproliferation and differentiation (73). The largest evaluation of anagrelide occurred in the Anagrelide Study Group trial, which enrolled 577 MPN patients with thrombocytosis. Of the 577 patients, 424 (73%) were treated with anagrelide for at least 4 weeks. Anagrelide treatment reduced the platelet count by 50% or to less than 600 x 10^9^/L for at least 28 days in 396 of the 424 (93%) evaluable patients. Thrombosis was not formally evaluated or reported in this study (74). In a more contemporary study of 79 ET patients (in addition to 18 patients with other MPNs and thrombocytosis) treated with anagrelide for 6 months, median platelet counts were significantly decreased from 743 x 10^9^/L to 441 x 10^9^/L after anagrelide treatment. The proportion of patients with a platelet count less than 600 x 10^9^/L increased from 30% to 77% after the 6‐month study period. In the 6-months prior to anagrelide treatment, 5% of patients had a major thromboembolic complication. However, during the 6 months of treatment the thrombotic complication rate was only 2%, consisting primarily of arterial thromboses. Notably, this study did not have a control group so it is unclear if this reduction was secondary to anagrelide or other factors (75).

Anagrelide has been directly compared to hydroxyurea for cytoreduction in ET in two different studies. In the United Kingdom Medical Research Council Primary Thrombocythemia 1 (UK-PT1) study, 809 patients with high risk ET (using PSVG criteria) were randomized to aspirin plus either hydroxyurea or anagrelide. High risk was defined as age over 60, current or previous platelet counts over 1,000 x 10^9^/L, a history of ischemia, thrombosis, or embolism, hemorrhage caused by ET, hypertension requiring treatment, or diabetes requiring treatment. After a median follow up of 39 months, patients treated with anagrelide were significantly more likely to experience the primary endpoint of arterial or venous thrombosis, hemorrhage, or death from any cause (odds ratio [OR]: 1.57; 95% CI: 1.04 to 2.37; p = 0.03). Anagrelide plus aspirin was also associated with an increased rate of arterial thrombosis (p=0.004) and serious hemorrhage (p=0.008) but a decreased rate of venous thrombosis (p=0.006). Transformation to myelofibrosis was significantly increased in the anagrelide arm (OR 2.92; 95% CI 1.24 to 6.86; p=0.01). The trial was stopped early (intended sample size was 560 per arm) by the data monitoring committee due to the differences observed. Notably, patients in the anagrelide group were more likely to discontinue therapy (76). The authors suggested that hydroxyurea plus aspirin should be considered as first-line therapy for high risk ET patients.

The ANAHYDRET study included 259 ET patients (using WHO criteria) who were previously untreated and deemed high risk if they had any of the following: older than 60 years, platelet count ≥1000 × 10^9^/L, increase of platelet count of more than 300 × 10^9^/L within 3 months, hypertension, diabetes, and/or a history of thrombo-hemorrhagic event. Patients were randomized to either hydroxyurea or anagrelide with an outcome of major and minor thrombotic and bleeding event. The protocol did not require mandatory concomitant medication with aspirin therapy and only 28.2% of patients were treated with low-dose aspirin. This study was designed *a priori* to determine non-inferiority between the two agents. The non-inferiority margin was chosen as a difference of 10 percentage points for a lower bound of the confidence interval for OR/HR of 0.40. After 760 patient-years of follow-up, there was no significant difference between major and minor arterial and venous thrombotic events, as well as severe and minor bleeding. Disease transformation into myelofibrosis or secondary leukemia was not observed did not differ between the two treatment groups. Thus, the study met its prespecified criteria for non-inferoirity (77). Differences in results for this trial as compared to the UK-PT1 trial previously mentioned may be due to timing of enrollment of patients (at diagnosis versus previously untreated), different diagnostic criteria used (PVSG-ET versus WHO-ET) and the restrictive use of aspirin.

Anagrelide has a unique toxicity profile that is important to recognize. In the Anagrelide Study Group trial, 24% of patients reported fluid retention and 2% were diagnosed with heart failure (76). In the ANAHYDRET study, hypertension, palpitations, and tachycardia were more common in the anagrelide group versus hydroxyurea (77).

## Future Directions

Primary prevention trials of thrombosis-related endpoints in PV and/or ET are difficult to conduct, requiring large accrual with long follow-up periods. Several of the randomized clinical trials discussed in this review were planned with much larger sample sizes in mind and stopped early due to low accrual and lack of feasibility to further accrue (18, 20). Thus, point estimates provided in these studies included wide confidence intervals due to the small number of events observed in these reduced cohort sizes. Declines in recruitment and competition with newer trials of promising agents are common. Targeting high-risk subjects to increase the expected event rates should be strongly considered when designing studies with thrombotic events as an endpoint of interest.

Biomarkers and other clinical endpoints have been explored for use as surrogate endpoints of thrombosis in order to provide earlier evidence of benefit (or lack of benefit). Capitalizing on design of studies around these surrogate endpoints is warranted. However, there is difficulty in knowing if the surrogate endpoint is meaningful in all populations. The relationship between a surrogate endpoint and the endpoint of interest should be well-established and not solely based on correlated data in order to be validated. Hematocrit control <45% could be a feasible surrogate endpoint for thrombosis given the results of the CYTO-PV study (20). The recent study by Rocca et al. that evaluated three different aspirin regimens in ET used a primary endpoint of TxA2, an unproven surrogate endpoint (68). However, this approach allowed measurable differences to be detected at two weeks instead of the years it would take for important outcomes such as thrombosis to occur.

Future studies should be aimed at validating surrogate endpoints for predicting thrombosis. One such method is trial-level validation, where the change in an endpoint is plotted against the change in a well-defined endpoint (i.e. thrombosis). The strength of this association is then measured by regression analysis. This strategy requires multiple trials that measure the same biomarker and endpoints (78). Expert consensus on potential biomarkers should be developed that are identified by biologic insights, feasibility, and generalizability across treatment modalities that can be incorporated in multiple trials in ET and PV to validate surrogate biomarker endpoints for thrombosis.

Complete response by ELN criteria is typically used as an endpoint in ET/PV trials, however thrombosis may not be associated with response. Some studies have seen a lack of association between response and thrombosis (14). The association between molecular responses achieved with a particular agent and the rate of thrombosis could be an extremely useful biomarker, but this relationship has yet to be validated. Future prospective RCTs should carefully consider measurement of response and its relationship to the development of thrombotic events.

Better data collection for thrombosis related events and adjudication of composite-related events is required. Of note, many studies combine arterial and venous thrombotic events. However, it is possible that certain therapies may have efficacy in reducing the burden of one type of thrombosis but not the other. Many of the trials discussed above used composite endpoints in order to have more events, thus reducing the required sample sizes. When using composite endpoints, each component should be clinically meaningful and success of the study should not be concluded if it is driven by the least meaningful components. Composite endpoints can “dilute” treatment effects seen as typically the most common (and usually not as clinically relevant) component of the composite drives the event rate. Careful choice of the individual items included in a composite endpoint is needed.

A non-inferiority trial design has possible use in this setting. This approach can exclude inferiority of one agent over another. A non-inferiority design requires consideration of the chosen non-inferiority margin which is very sensitive to the assumed effect of the new treatment relative to the past performance of the comparator arm. FDA guidance exists for choosing this margin of interest. Both intent-to-treat and per-protocol analyses should be reported in a non-inferiority study as an intention-to-treat analysis may be biased toward a false-positive conclusion of non-inferiority due to a narrowing of the difference between treatments if considerable amounts of non-adherence, cross-over or loss to follow-up occurs.

Continued emphasis should be placed on encouraging standardized and pooled data collection across RCTs for the purposes of meta-analyses. However, there is wide variability in patient demographics, clinical characteristics and treatment practices (i.e. hematocrit control) across geographical regions. Multi-institutional studies can lead the way in standardization and incorporation of biomarkers for use as thrombosis endpoints and to create uniform data collection systems that would serve as a model for future investigator-initiated and industry-sponsored clinical trials in MPNs.

Despite thrombosis accounting for the majority of morbidity and mortality in PV and ET, clinical research up to this point has been largely unregimented when it comes to evaluating the impact of therapeutic strategies on thrombosis. As such, we are unable to recommend on cytoreductive therapy over another for primary prevention of thrombosis in MPN patients. While many disease-specific limitations exist to account for the lack of attention toward thrombosis reduction, we believe these obstacles can be overcome with surrogate endpoint and biomarker validation studies, in addition to uniform clinical trial design. Attention by both investigators and regulatory agencies on effective methodology to measure the impact of thrombosis is urgently needed in order to advance MPN research and provide longer and healthier lives for patients with PV and ET.

## Author Contributions

All authors listed have made a substantial, direct, and intellectual contribution to the work, and approved it for publication.

## Conflict of Interest

The authors declare that the research was conducted in the absence of any commercial or financial relationships that could be construed as a potential conflict of interest.
